# Vapor Responsive One-Dimensional Photonic Crystals from Zeolite Nanoparticles and Metal Oxide Films for Optical Sensing

**DOI:** 10.3390/s140712207

**Published:** 2014-07-09

**Authors:** Katerina Lazarova, Hussein Awala, Sebastien Thomas, Marina Vasileva, Svetlana Mintova, Tsvetanka Babeva

**Affiliations:** 1 Institute of Optical Materials and Technologies “Acad. J. Malinowski”, Bulgarian Academy of Sciences, Acad. G. Bonchev str., bl. 109, 1113 Sofia, Bulgaria; E-Mail: marina@iomt.bas.bg; 2 Laboratoire Catalyse & Spectrochimie, Université de Caen, 6, boulevard du Maréchal Juin, 14050 Caen Cedex, France; E-Mails: hussein.awala@ensicaen.fr (H.A.); sebastien.thomas@ensicaen.fr (S.T.); svetlana.mintova@ensicaen.fr (S.M.)

**Keywords:** one-dimensional photonic crystals, nanosized zeolites, sol-gel Nb_2_O_5_, multilayered structures, optical sensors

## Abstract

The preparation of responsive multilayered structures with quarter-wave design based on layer-by-layer deposition of sol-gel derived Nb_2_O_5_ films and spin-coated MEL type zeolite is demonstrated. The refractive indices (n) and thicknesses (d) of the layers are determined using non-linear curve fitting of the measured reflectance spectra. Besides, the surface and cross-sectional features of the multilayered structures are characterized by scanning electron microscopy (SEM). The quasi-omnidirectional photonic band for the multilayered structures is predicted theoretically, and confirmed experimentally by reflectance measurements at oblique incidence with polarized light. The sensing properties of the multilayered structures toward acetone are studied by measuring transmittance spectra prior and after vapor exposure. Furthermore, the potential of the one-dimensional photonic crystals based on the multilayered structure consisting of Nb_2_O_5_ and MEL type zeolite as a chemical sensor with optical read-out is discussed.

## 1. Introduction

Photonic crystals (PhCs) are artificial structures where media with two different refractive indices are arranged in a periodic manner with the period comparable to the wavelength of light in the visible and near infrared spectral ranges (500–1500 nm) [1,2]. Owing to the phenomenon of constructive interference certain stop bands open when the PhCs are probed with light incident at any direction. Under particular conditions the stop bands for different directions overlap and for the entire wavelength range light propagation is forbidden for all directions and polarization of incident light. The band of forbidden wavelengths is commonly referred to as “complete or 3D photonic band gap”.

One-dimensional PhCs (1D-PhCs) could be realized through alternative stacking of low- and high-refractive index layers [3]. When the PhCs' optical thicknesses (the product of refractive index and physical thickness) equal a quarter of the operating wavelength, then the structures are called Bragg stacks, or Bragg reflectors. For both 1D-PhCs and Bragg stacks a one-dimensional photonic band gap exists that exhibits a blue shift with increasing incident angle. If the stop band is positioned in the visible spectral range a strong coloration referred as “structural” color is observed. The position of the reflectance band and color depend strongly on the optical thickness of the constituent materials. Thus 1D-PhCs could be controlled by the influence of external stimuli, for example gas vapors. In this way, responsive one-dimensional structures for optical sensing applications can be prepared. This concept has already been demonstrated for multilayered structures comprising mesoporous TiO_2_ and SiO_2_ [4], TiO_2_ and synthetic Laponite [5,6], TiO_2_ and SiO_2_ nanoparticles [7,8], SiO_2_ and SnO_2_, SiO_2_ and Sb-doped SnO_2_ [9], zeolite nanoparticles and TiO_2_ and ZnO particles [6] or Ta_2_O_5_ films [10], polymeric hydrophobic and hydrophilic layers [11]. The main disadvantage of these Bragg stacks is the dependence of their reflectance bands on the direction of incident light, resulting in structural color changes at different viewing angles. It is obvious that this will be a problem when visual inspection of color is used as a detection approach. One possible solution is to prepare structures with omnidirectional reflection that have high reflectance for all directions and polarization of light. In this case one and the same color will be observed at all viewing angles. Through inspection of calculated omnidirectional bandwidth as a function of both high (*n_H_*) and low (*n_L_*) refractive indices in the Bragg stack, it was shown that for certain conditions, it is possible for an omnidirectional reflectance to be achieved for Bragg stacks [12]. Specifically, a refractive index *n_H_* higher than 2.264, and an optical contrast (*n_H_*-*n_L_*) higher than 0.75 are the two conditions [12]. Additionally at fixed *n_H_* there is *n_L_* value below which omnidirectional reflectance could not be achieved [12]. Recently we have shown that using materials with high optical contrast such as GeSe_2_ (*n_H_* = 2.65) and zeolite nanocrystals (*n_L_* = 1.19) it is possible for an omnidirectional reflectance to be obtained for Bragg stacks even using zeolites with very low *n_L_* [13]. However finding porous materials with high refractive index is not a trivial task. To overcome this problem, the preparation of quasi-omnidirectional (q-ODR) PhCs exhibiting high reflectance for all polarization but in a narrower range of incident angles as compared to omnidirectional PhCs is considered.

In this study we demonstrate the preparation of one-dimensional photonic crystals based on MEL type zeolite and Nb_2_O_5_ for sensing applications. MEL type zeolite nanocrystals are used for both the sensing and transducing elements, while a sufficient optical contrast is ensured by sol-gel derived Nb_2_O_5_ film [14]. Nb_2_O_5_ is a high refractive index material and could be assembled in thin film form by simple methods [14]. Besides, recently we have reported that Nb_2_O_5_ could be successfully used as a high refractive index building block of a vapor responsive Bragg stack that comprises MFI zeolite as sensing element [15]. Zeolites are crystalline materials with framework-type structures built of regular and uniform pores of molecular dimensions [16]. The change of thickness and/or refractive index of the films (layers) based on zeolite nanocrystals are due to sorption, ion-exchange and capillary condensation of molecules adsorbed in their pores [17,18]. The particular choice of MEL zeolite was made considering their hydrophobicity and high sensitivity towards acetone [10]. The MEL type zeolite is a pure silica material with three-dimensional channels with a pore opening of 0.53 × 0.54 nm. The pure silica form of the MEL zeolite leads to increased hydrophobicity in contrast to the alumina loaded structures. For the preparation of multilayered structure, such as one-dimensional crystal, it is important to use a simple, inexpensive and reproducible technique to deposit thin layers of zeolites and Nb_2_O_5_. The spin coating method is considered as simple and compatible with different surfaces. Additionally, it allows effective control of film thicknesses by varying concentration of coating suspensions and the spin-on conditions [19,20].

In this study the preparation of vapor responsive one-dimensional photonic crystals consisting of MEL type zeolite and Nb_2_O_5_ films is reported. The omnidirectional reflectivity is probed through reflectance measurement at normal and oblique incidence of unpolarized and s- and p-polarized light. The potential application of the multilayered structures as chemical sensors with optical read-out working is demonstrated by measuring the sensor response to acetone/argon cycling switching.

## 2. Materials and Methods

The Nb sol was prepared by a sonocatalytic method using NbCl_5_ (99%, Aldrich, St. Louis, MO, USA) as a precursor according to the recipe in [14,21]: NbCl_5_ (0.400 g) was mixed with ethanol (8.3 mL, 98%, Sigma-Aldrich) and distilled water (0.17 mL). The solution was subjected to sonication for 30 min and aged for 24 h at ambient conditions prior to spin coating [14]. Thin Nb_2_O_5_ films were deposited by dropping of 0.3 mL of the coating solution on both the pre-cleaned Si substrates and pre-cleaned optical glass plates, then spinning at a rate of 2500 rpm for 30 s was carried out. After deposition, the films were annealed in air at 320 °C for 30 min (10 °C/min heating rate).

Nanosized MEL zeolite crystals were synthesized from a colloidal precursor suspension with the following chemical composition: 1.0:0.3:4.0:18.0 SiO_2_-TBAOH-EtOH-H_2_O (where TBAOH-tetrabutylammonium hydroxide, EtOH-ethanol). The precursor suspension was heated at 90 °C for 68 h [22]. After the synthesis, the precursor suspension was purified using high-speed centrifugation, and finally redispersed in ethanol to obtain a coating suspension with a solid concentration of 5 wt. %. The hydrodynamic diameter of the MEL zeolite crystals measured by DLS was 90 nm. The MEL zeolite-ethanol suspension was spin-coated on silicon wafer (2500 rpm and 30 s), followed by annealing at 320 °C for 30 min.

Multilayered structures comprising two, three, five and seven layers were prepared by alternative deposition of Nb_2_O_5_ and MEL zeolite films with target thicknesses of 55 nm and 105 nm, respectively on glass substrate. The target thicknesses of both films were calculated aiming at their optical thicknesses—the product of physical thickness and refractive index, to be a quarter of the operating wavelength, *i.e.*, 500 nm in the current case. In order to achieve the correct thicknesses of the films, the concentrations of the zeolite suspension and Nb sol were optimized by addition of appropriate amount of solvent-ethanol [10,14]. An annealing of the films at 320 °C for 30 min was applied after deposition of the MEL nanozeolite. Finally the structures were subjected to calcination at 320 and 450 °C for 30 min.

The surface and crosssection views of the multilayered structures were studied by Scanning Electron Microscopy (SEM) using a JEOL JSM6700F SEM (accelerating voltage of 30.0 kV, JEOL, Tokyo, Japan). Reflection spectra (R) of zeolite films were measured in the spectral range 320–900 nm using a UV-VIS-NIR spectrophotometer (Cary 5E, Varian, Australia) at normal and oblique incidence of unpolarized and p- and s-polarized light. Refractive index (*n*), extinction coefficient (*k*), and thickness (*d*) of the films were determined simultaneously from the reflection measurements using non-linear curve fitting method described in details elsewhere [10,14]. The experimental errors for the three parameters, *n*, *k* and *d* are 0.005, 0.003 and 2 nm, respectively.

**Figure 1.**
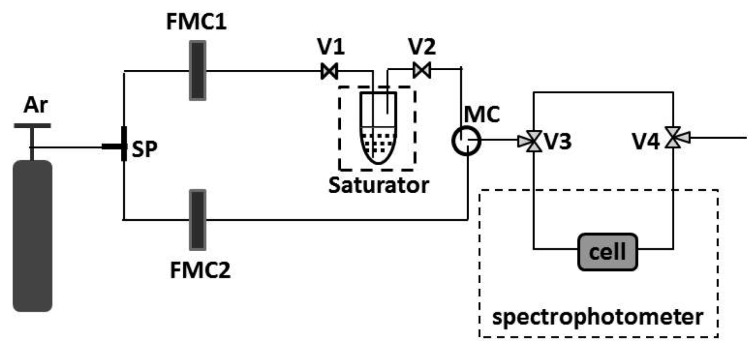
Bubbler system for generation of acetone vapor; SP—splitter, FMC1 and FMC2—flow mass controllers, V1 and V2—two path valves, V3 and V4—three path valves, MC—mixing chamber.

The vapor sensing measurements on films were implemented in a Cary 5E spectrophotometer equipped by a homemade bubbler system for generation of vapors from liquids as shown in Figure 1. Argon is used as carrier gas for going through the splitter (SP) and two flow mass controllers (FMC1) and (FMC2) with full-scale range of 10 and 1000 sccm, respectively. After FMC1 the carrier gas is utilized to bubble through liquid acetone in the saturator. The saturator is thermally isolated and kept at constant temperature. The flow rate of the bubbling gas is sufficiently low in order to ensure that the gas concentration reaches the state of saturation. The carrier gas (from the saturator) and the diluting Ar gas from FMC2 are mixed in the mixer chamber (MC), and directed into the cell located in the spectrophotometer or out of the system through valves V3 and V4. The concentration of acetone in the range from 100 to 93,000 ppm or relative pressure *p*/*p*_0_ (*p*_0_ pressure of saturated vapors) from 0 to 1 are controlled by tuning the flows from FMC1 and FMC2 and keeping the saturator at temperature of 0 °C. Transmittance spectra of the samples were measured before and after exposure the films to the vapors using a Cary 5E spectrophotometer with an accuracy of 0.1%. Prior to the measurements all films were exposed to Ar vapor for 10 min. In this case the valves V1 and V2 are turned out and only Ar gas from FMC2 reaches the cell.

## 3. Results and Discussion

### 3.1. Morphology Study of Photonic Crystals Based on Nb_2_O_5_ and MEL Zeolite Layers

**Figure 2.**
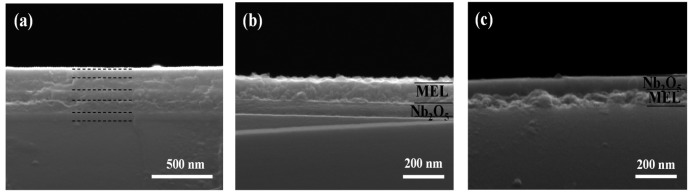
SEM cross-section image of (**a**) 5-layers photonic crystals of alternating Nb_2_O_5_ and MEL zeolite films—the Nb_2_O_5_ film is the first layer deposited on the Si-substrate; (**b**) MEL zeolite film is on top of the Nb_2_O_5_ film; and (**c**) Nb_2_O_5_ film on top of MEL zeolite film.

Figure 2 shows the surface morphology and cross-sectional view of five-layered and double-layered structures consisting of Nb_2_O_5_ and MEL type zeolite. In order to obtain high reflectance of the photonic crystals, the film close to the substrate and in the top of the structure is chosen to be the high refractive index Nb_2_O_5_ film. The several alternating layers are well seen in Figure 2a.

**Figure 3.**
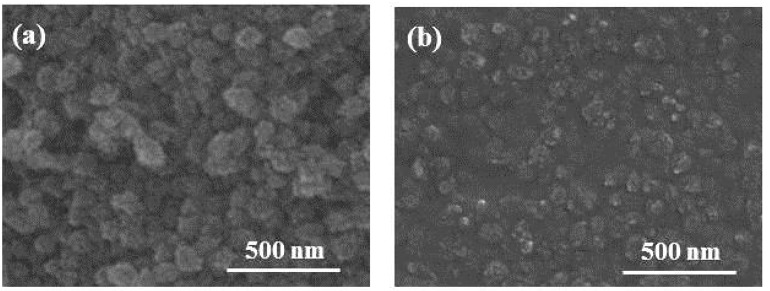
Surface morphology of (**a**) single MEL zeolite film deposited on Si-substrate and (**b**) PhC consisting of five layers of alternating Nb_2_O_5_ and MEL zeolite films.

More clearly, the very smooth Nb_2_O_5_ film featureless is visible in Figure 2b. A distinct interface exists between the Nb_2_O_5_ and the MEL zeolite films deposited on top of it (Figure 2b). Besides the MEL type zeolite nanocrystals are well packed in a uniform film that covers the surface of the entire substrate Figure 2b. However when the Nb_2_O_5_ film is deposited on top of the MEL zeolite film, some interpenetration between the films is observed (Figure 2c), which is the reason of lack of clear boundary between them. The surface morphology of single MEL zeolite film and 5-layered PhC on silicon substrates is studied by SEM (Figure 3). Considering that the Nb_2_O_5_ film is very smooth and featureless, one can see that the MEL zeolite crystals mainly are responsible for the surface roughness of the oxide-zeolite structures (PhC). However, the surface roughness can be decreased by depositing the Nb_2_O_5_ film on top of the PhC structure (Figure 3).

### 3.2. Optical Properties of Photonic Crystals Based on Nb_2_O_5_ and MEL Zeolite Layers

**Figure 4.**
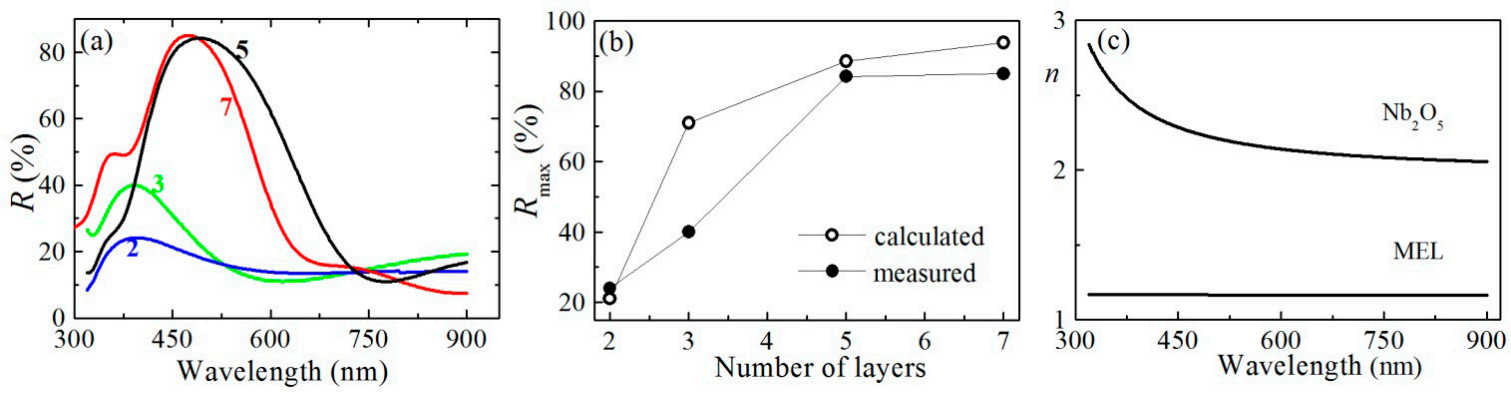
(**a**) Reflectance spectra at normal incidence of unpolarized light for PhC structures comprising of 2, 3, 5 and 7 layers of alternating Nb_2_O_5_ and MEL zeolite films deposited on glass substrate. In all samples the film close to the substrate is Nb_2_O_5_; (**b**) measured and calculated values of maximum reflectance as a function of number of the layers in the stack; and (**c**) dispersion curves of refractive index for Nb_2_O_5_ and MEL zeolite films.

The development of reflectance band with increasing the number of Nb_2_O_5_ and MEL zeolite layers in the photonic structures is studied by measuring the reflectance spectra (*R*) at normal incidence of unpolarized light; the structures comprising of two, three, five and seven layers of alternating Nb_2_O_5_ and MEL zeolite films are presented in Figure 4a.

For all photonic structures, the film close to the glass substrate is Nb_2_O_5_. It is seen that *R* increases with increasing the number of layers in the structures, and the *R* reaches a value of 85% for the 7-layers PhCs. The position of the reflectance band measured is 474 nm, which is close to the target value of 500 nm. The small difference is probably due to the deviations in the thickness of the Nb_2_O_5_ film. The Nb_2_O_5_ film deposited is thinner as compared to the calculated one due to the partial penetration in the zeolite film as shown in Figure 2c. Accordingly, the decrease of the optical thickness induces a blue shift of the reflectance band. The comparison between the calculated and measured values of maximum reflectance is presented in Figure 4b. The measured values are lower than the calculated ones since the PhC differs from the theoretically predicted films with smooth boundaries having the target value thicknesses of 55 nm and 105 nm.

**Figure 5.**
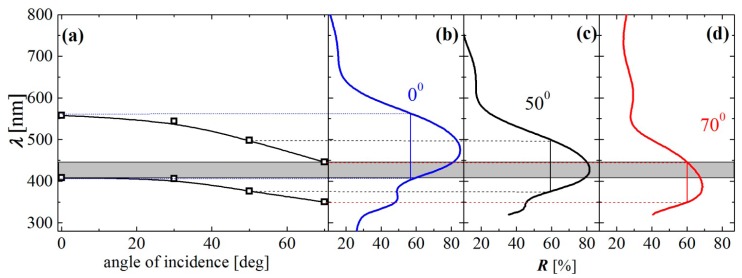
(**a**) Measured reflection band edges for *R*_pol_ = (*R*_p_ + *R*_s_)/2 at levels of 60% as a function of the incident angle. Measured reflectance spectra *R*_pol_ of PhC comprising 7 alternating layers of Nb_2_O_5_ and MEL zeolites as a function of wavelength at incident angles (**b**) 0°, (**c**) 50° and (**d**) 70°. The shaded area represents the quasi-ODR band for the angular range 0°–70°.

Further, refractive index (*n*) and thickness (*d*) of the films were calculated using the reflectance spectra measured and the non-linear curve fitting method [14]. Figure 4c shows the dispersion curves of refractive index of the Nb_2_O_5_ and the MEL zeolite films with thicknesses of 55 and 105 nm, respectively. Because both films are transparent in the studied spectral range, they obey normal dispersion, *i.e.*, the *n* decreases with wavelength. For wavelength of 500 nm the refractive index values of Nb_2_O_5_ and MEL films are 2.22 and 1.17, respectively, that means an optical contrast of 1.05 is achieved. Considering the high optical contrast achieved along with the high refractive index values of the Nb_2_O_5_ film, we could expect omnidirectional band to be open up. To verify this assumption, the reflectance spectra of the photonic crystals are measured at different incident angles in the range 0°–70° for both *p*- and *s*-polarized light. Figure 5 shows the measured reflection band edges for *R*_pol_ = (*R*_p_ + *R_s_*)/2 at levels of 60% as a function of the incident angle (Figure 5a), and the measured spectra of *R*_pol_ at incident angles of 0°, 50° and 70° (Figure 5b–d).

Generally, the omnidirectional (ODR) band is defined between the short reflectance edge at 0° and long reflection edge at 90° [12]. It is seen from Figure 5a that the quasi ODR band exists and it is defined between the short reflectance edge at 0° (410 nm) and the long reflection edge at 70° (444 nm). The quasi-omnidirectional (q-ODR) band is centered at wavelength of 427 nm, and it has a width of 34 nm and reflectance values higher than 60%. This means that the reflectance of the PhC within the spectral range 410–444 nm is higher than 60% for all incident angles in the range 0°–70°. Analogically a quasi ODR band with higher reflectance (*R* > 80%) is centered at 445 nm but the bands width is only 2 nm.

### 3.3. Sensing Properties of Photonic Crystals Based on Nb_2_O_5_ and MEL Zeolite Layers

**Figure 6.**
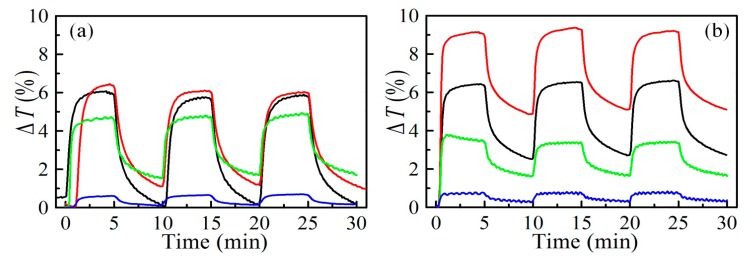
Kinetics of transmittance changes for a sensor comprising two (blue line), three (green line), five (black line) and seven (red line) alternating Nb_2_O_5_ and MEL zeolite films annealed at 320 °C (**a**) and 450 °C (**b**) within 3 cycles of adsorption and desorption of acetone at room temperature.

The adsorption/desorption experiments of acetone with photonic crystals based on different number of Nb_2_O_5_ and MEL zeolite layers annealed at 320 and 450 °C are carried out, and the results are depicted in Figure 6. The transmittance signal (Δ*T*) was measured as a function of time, and the measurement is repeated for three cycles of adsorption/desorption. The particular wavelength of measurements is selected separately for each sample by measuring spectra prior and after acetone exposure. Then the wavelength of the biggest change in the spectra was chosen for measurements. It was revealed that only the short wavelength edge (the blue edge) of the photonic band shifts. There is no significant change of the red edge. Thus the spectral position of the measurement was chosen to be at wavelength of 350 nm, 360 nm, 402 nm and 410 nm for two, three, five and seven layers, respectively. Small variations of the wavelength within the area of photonic band edge do not lead to significant difference in the measured signal. The experiments were conducted at room temperature with samples preliminary treated at two temperatures. The influence of the number of layers in the PhC structure on the strength of transmittance changes is clearly seen: Δ*T* changes from 0.6% to 6.5% and from 0.7% to 9% in the case of sample annealed at 320 °C and 450 °C, respectively, when the number of layers increases from 2 to 7.

The exposure of the PhC to acetone results in vapor condensation in the pores and replacement of the air inside with acetone with higher refractive index. As a result the effective refractive index of the structure increases thus leading to red shift of the stop band edges (*i.e.*, transmittance band edges) and increase of *T* at a fixed wavelength. In particular as stated in [23] changes in transmittance or reflectance provide a mean to identify the preferential adsorption in one specific type of layer in the structure. The preferential solvent condensation within the zeolite pores would lead to reduction of optical contrast and narrowing of photonic band. On the contrary, preferential adsorption onto Nb_2_O_5_ pores would increase the optical contrast hence shifting the stop band and widening it. The observed narrowing of the stop band in our case (shift of blue edge without significant change of the red one) means that only the refractive index of the zeolite film changes when the structure is exposed to acetone vapors. This may be regarded as an advantage because the selectivity of zeolite toward adsorption of different gases could be fully explored.

Both adsorption and desorption branches comprise two clearly distinguishable processes (Figure 6). After switching on the acetone vapor a fast adsorption takes place more pronounced for samples treated at 450 °C, followed by steady state signal. Analogically after switching off the acetone a very fast desorption takes place followed by the slower process. Besides, at 320 °C the desorption is almost completed within 5 min after the acetone is switched off, while for the stacks annealed at higher temperature longer time is needed for returning into the initial state. Therefore we can assume that the adsorption for structures annealed at 450 °C is faster but desorption is slower as compared to sample thermally treated at 320 °C. To check this and study the adsorption and desorption processes for different number of layers and for the two temperatures of annealing, we calculated the time constants for four processes described above.

Purposely we assumed that *T* changes with time according to the following exponential dependence:

(1) $$T=T_{0}+Ae^{\pm \frac{t}{t_{0}}}$$

where *T*_0_ is the initial value of transmittance, *A* is a constant, *t* is time and *t*_0_ is the time constant of the process. The sign is plus for the adsorption process (*T* increases) and minus for desorption process when *T* decreases.

If we apply logarithmic operation to Equation (1), then it can be rewritten in the form:

(2) $$ln\left(T-T_{0}\right)=A\pm \frac{t}{t_{0}}$$

The slope of the linear fit of the plot *ln*(*T*−*T*_0_) *versus t* gives the time constant 1/*t*_0_.

**Figure 7.**
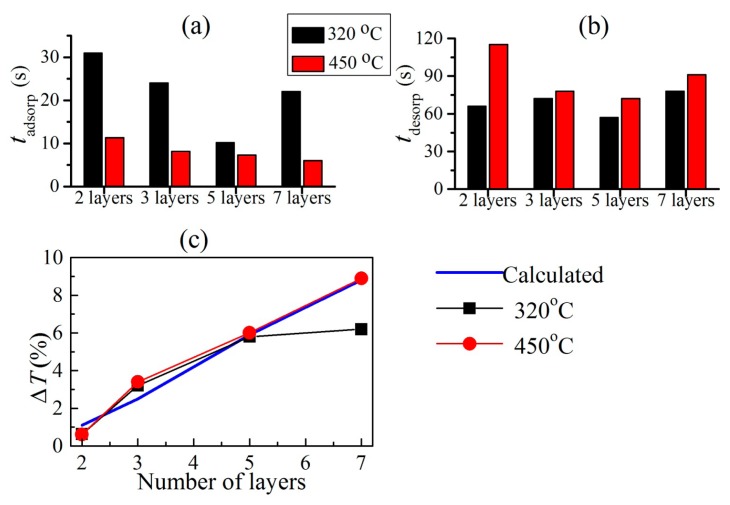
Time constants of adsorption (**a**) and desorption (**b**) of photonic structures comprising different number of layers annealed at 320 °C and 450 °C; and (**c**) calculated and measured changes of *T* as a function of number of the layers in the structures annealed at 320 °C and 450 °C.

Figure 7 presents the time constants for fast adsorption and desorption in photonic structures comprising different number of layers (two, three, five and seven) annealed at both temperatures, *i.e.*, 320 and 450 °C. The comparison between the structures annealed at different temperatures shows that adsorption is faster for all samples preliminarily treated at 450 °C. Desorption times are almost equal with exception of the 2-layers stack where the preliminary annealing at 320 °C is favorable for the quickest desorption.

The calculated values Δ*T_calc_* (= *T_bef_* − *T_aft_*) of transmittance of the structures before (*T_bef_*) and after (*T_aft_*) the vapor exposure are plotted in Figure 7c as a function of the number of layers in the PhC structure. For calculation of *T_bef_* and *T_aft_* the matrix transfer method [24] is used along with the assumption that all zeolite films in the particular structure contribute to *T_aft_*. Preliminary sensing measurements of Nb_2_O_5_ films have shown that the *n*, *k* and *d* for Nb_2_O_5_ films are the same before and after exposure to acetone. The values of *n*, *k* and *d* of MEL films before and after exposure to acetone were determined from nonlinear curve fitting of reflectance spectra of single films deposited on Si substrate [14]. In the current case, it is seen that Δ*T_calc_* gradually increases from 1.1% to 8.8% with the number of layers in the structure. The measured changes Δ*T_meas_* for sample treated at 320 and 450 °C are also presented in Figure 7c. It is seen that Δ*T_meas_* increases from 0.6% to 4.7% and to 6% for two, three and five layers (annealing at 320 °C), respectively, but there is no further increase of Δ*T_meas_* for seven layers. In contrast for samples treated at higher temperature (annealed at 450 °C) the Δ*T_meas_* gradually increases with the number of layers reaching value of 8.9% for the 7-layered PhC structure.

The comparison between measured and calculated changes shows that Δ*T_calc_* coincides very well with Δ*T_meas_* for all samples annealed at 450 °C. On the contrary for samples annealed at 320 °C there is a difference between Δ*T_calc_* and Δ*T_meas_* for the 7-layers PhC structure. Considering that calculations are made with an assumption that all MEL films contribute to the change of *T*, we made the conclusion that at 450 °C all layers contribute to the transmittance changes. While at 320 °C only five layers are accessible to the analyte (acetone in the current case).

We can speculate that the higher temperature of annealing induces some porosity in the Nb_2_O_5_ film and the acetone molecules more easily penetrate throughout it. A possible reason for the enhanced porosity of the Nb_2_O_5_ film could be its structural transformation from amorphous state at 320 °C to crystalline one at 450 °C [25].

## 4. Conclusions

MEL zeolite and sol-gel Nb_2_O_5_ films are used successfully as building blocks of vapor responsive one-dimensional photonic crystal structures. It is demonstrated that the obtained difference in refractive indices of the films of more than 1 is sufficient for wide quasi-omnidirectional reflectance band (q-ODR) to be developed. With physical thicknesses for Nb_2_O_5_ and MEL zeolite films of 55 and 105 nm, respectively, a q-ODR band centered at 427 nm with a width of 34 nm and reflectance value higher than 60% is achieved in the angle range 0°–70°.

The potential application of one-dimensional photonic crystals as optical read-out vapor sensors is demonstrated by preparation of structures of alternating Nb_2_O_5_ and MEL zeolite films comprising different number of layers with quarter-wavelength optical thickness; the transmittance spectra prior and after exposure to acetone vapor are measured. Changes in *T* from 0.6% to 6.5% and from 0.7% to 9% in the case of PhC structures annealed at 320 °C and 450 °C, respectively are obtained when the number of layers is increased from two to seven. Besides the comparison between the calculated and measured signals reveals that all layers contribute to the transmittance changes for samples calcinated at 450 °C, while at 320 °C only five layers are accessible to the analyte. Considering that temperature annealing at 450 °C induces structural transformation in the Nb_2_O_5_ film, the assumption is made that the preliminary annealing of samples at 450 °C favors higher porosity to be achieved facilitating in this way the penetration of acetone vapors throughout the film. The calculated time constants show that the fastest adsorption of 6 s occurs for a 7-layers PhC subjected to preliminary treatment at 450 °C.
